# Long non-coding RNA RP11-379k17.4 derived microRNA-200c-3p modulates human endometrial cancer by targeting Noxa

**DOI:** 10.7150/jca.51023

**Published:** 2021-02-22

**Authors:** Weijuan Xin, Xiaodong Gao, Peng Zhao, Taiyong Wang, Xue Ding, Qianyu Wu, Keqin Hua

**Affiliations:** 1 Department of Obstetrics and Gynecology, Obstetrics and Gynecology Hospital of Fudan University, 128 Shen-Yang Road, Shanghai 200090, China.; 2 Department of Obstetrics and Gynecology, Shanghai East Hospital, Tongji University School of Medicine, 150 Jimo Road, Pudong New Area, Shanghai 200120, China.; 3 Department of Internal Medicine, People's Hospital of Dezhou, 1751 Xinhu Street, Dezhou 253001, China.; 4 Department of Oncology, People's Hospital of Dezhou, 1751 Xinhu Street, Dezhou 253001, China.

**Keywords:** lncRNA RP11-379k17.4, endometrial cancer, microRNA-200c-3p, noxa, ceRNAs

## Abstract

**Objective:** The research paid close attention to the function of lncRNA-related endogenous competitive RNAs (ceRNAs) network in endometrial cancer (EC).

**Methods:** 45 primary endometrial cancer tissues (EC) and 45 normal endometrium (NE) were included in the research. The online software StarbaseV2.0 was made use of forecasting the lncRNA which most likely contained microRNA-200c-3p combining sites and could interact with microRNA-200c-3p. Subsequently, we chose lncRNAs which were consistent with the characteristics of polyadenylation of lncRNAs and lower expression in EC than that of NE. After that, lncRNAs, which were related with the microRNA-200c-3p-noxa network, were identified.

**Results:** Rp11-379k17.4, a new gene related to endometrial cancer, was identified as noncoding RNA. It was a more effective ceRNA associated with the microRNA-200c-3p-noxa network.

**Conclusion:** LncRNAs possess microRNA response elements (MREs) and give scope to significant roles in the post-transcriptional mechanism in EC.

## Introduction

Endometrial cancer (EC) is one of the most common female cancers in developed countries 1. EC is the third common malignant tumor of the feminine reproductive system in China, and its morbidity is increasing year by year 2. Studies have shown that the 5-year overall survival rate (OS) of early (FIGO I-II) EC patients is 74-91%, while the 5-year survival rates of stage III and IV sufferers are only 57%-66% and 20%-26% 3. Relapse occurs in 10-20% of early (I-II) and 50-70% of late (III-IV) patients after initial treatment 4. Therefore, there is an urgent need to explore biomarkers in order to adapt different treatment regimens, identify high-risk patients with recurrence, guide postoperative treatment, and determine follow-up regimens.

Noxa is also known as APR (alt-derived PMA-responsive gene) or PMAIPl (phorbol-12-mystate-13-induced protein 1) 5. Noxa is a pro-apoptotic member of the bcl-2 family and a downstream target gene of p53 6, which does not only produce an effect in p53-mediated apoptosis 7, but also induces gene expression through p53 independent pathways to promote apoptosis 8-12. Noxa mRNA contains a microRNA combining site in the 3'UTR domain. The microRNA combines to the 3'UTR target of noxa, thereby affecting cell apoptosis. For example, in colorectal cancer, mir-1271-5p regulates noxa expression by combining to the 3'UTR of noxa 13, and microRNA-200c-3p inhibits the expression of noxa by combining with the 3'UTR target of noxa, so as to suppress apoptosis 14. However, noxa has not been studied in endometrial cancer.

Long non-coding RNA is a class of RNA molecules whose transcript length exceeds 200 nucleotides 15. By regulating methylation, modifying histones, remodeling chromatin, and transcription-related microRNAs 16,17, it can affect gene transcription and the post-transcriptional regulatory process, and regulate the splicing, translation, degradation and stability of mRNA. Through these ways, it can participate in the process of cell growth, apoptosis and tumorigenesis of various tumors 18,19. In 2011, Salmena 20 proposed the hypothesis of competitive endogenous RNA (ceRNA). It describes a complex post-transcriptional horizontal regulate and control network in which lncrnas can act as natural miRNA sponges to inhibit the function of other RNAs by co-competing for one or more miRNA binding sites.

MicroRNA (miRNA) is a kind of noncoding small RNAs with regulatory functions, consisting of about 22 nucleotides, which can bind to target mRNA through complete or incomplete pairing with 3' non-translation regions to regulate post-transcriptional expression of genes 21. Several studies have shown that microRNA-200c-3p is obviously increased in endometrial cancer 22. MiR-200c identified the AP-2α gene 3'UTR, negatively regulating the expression of endogenous AP-2α protein and enhancing the sensitivity of cisplatin 23. However, it is not clear whether microRNA-200c-3p can affect cell apoptosis in endometrial carcinoma though binding to noxa's 3'UTR target site.

In this study, we identified one novel gene RP11-379k17.4 related with EC and proven to be noncoding RNA. It was a more effective ceRNA associated with the microRNA-200c-3p-noxa network.

## Materials and Methods

### Subjects

In this research, 45 EC and 45 NE endometrium were included. All the endometrial tissues and ethical approval were from the Obstetrics and Gynecology Hospital of Fudan University, Shanghai, China. NE came from women who had a hysterectomy for unrelated endometrial diseases, such as fibroid or prolapse. Inclusion criteria: none of the patients had received preoperative chemotherapy, radiotherapy or hormone therapy and all had signed informed consent. Exclusion criteria: patients who had received hormone or chemoradiotherapy before surgery or did not sign informed consent.

### Prediction and Recognition of LncRNA

The microRNA-200c-3p and its objective lncRNA were predicated by bioinformatics. According to the objective gene, there are conservative or non-conservative sites matching the candidate miRNA seed region, forming RNA secondary structure. The lncrna for microRNA-200c-3p was predicated by Starbase V2.0 software. The level of lncRNA expression was normalized to that of 5S rRNA, and the relative expression was counted by comparative CT.

### Quantitative Real-Time Polymerase Chain Reaction

Trizol reagent was used to separate total RNA from the endometrial tissues, and nanodrop-1000 spectrophotometer (Wilmington) was used to evaluate its quality and quantity. Real-time PCR was carried out with 2×SYBR Premix Ex TaqTM mixed reagents (Takara, Dalian, China). The expression levels of mRNA and miRNA were measured by the Bio-systems 7500 system using GAPDH and 5S rRNA for normalization, respectively. A primer designing software package was used for the primers (microRNA-200c-3p forward: 5'-CGCGTAATACTGCCGGGTAAT; reverse: 5'-AGTGCAGGGTCCGAGGTATT. NOXA forward: 5'-AAGAACGCTCAACCGAGCC; reverse: 5'-TGCCGGAAGTTCAGTTTGTCT).

### Western Blot Analysis

The total protein was separated from the endometrial tissues, centrifuged at 4 °C, 10,000×g for 10 min. 30 ug of the total protein was analyzed by western blot and transferred to PVDF membrane (Millipore, USA). Subsequently the membranes were incubated with the antibodies: NOXA (1:1000; Cell Signaling Technology, Inc.; cat. no. #14766) and GAPDH (1:1000; Cell Signaling Technology, Inc.; cat. no. #5174). The second antiserum and immunoreactive protein were checked by ECL.

### Luciferase Assay

Using Lipofectamine 3000 with microRNA-200c-3p mimic or inhibitor into HEK293 cells to co-transfect PmirGLO-lncRNA-wild-type (lncRNA-wt) and PmirGLO-lncRNA-mutant (lncRNA-mut) at a ratio of 50:1. In light of the manufacturer's protocol, luciferase activities were determined using the dual-luciferase assay kit (Promega, Madison, WI, USA) after 48 h of transfection. Firefly luciferase activity was standardized to the renilla luciferase activity.

### Statistical Analysis

SAS version 8.2 software was sued to the data analyses. All the data are revealed as the means ± standard deviation (SD) from three experiments. Using the two-tailed Students' t-test for comparisons between two groups or analysis of variance (ANOVA) followed by post hoc pairwise multiple comparisons among several groups to statistically analyze. A value of p < 0.05 was regarded as statistically significant.

## Results

### Identification of endogenous lncRNA for microRNA-200c-3p in EC

**Figure 1:**
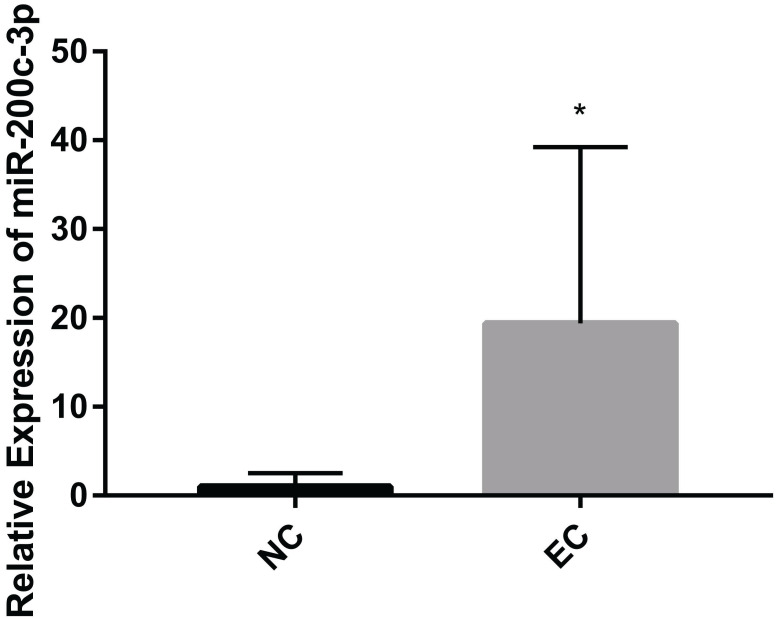
The relative expression levels of microRNA-200c-3p in EC tissues (*P<0.05). microRNA-200c-3p significantly increased in EC tissues as compared to NE tissues.

**Figure 2:**
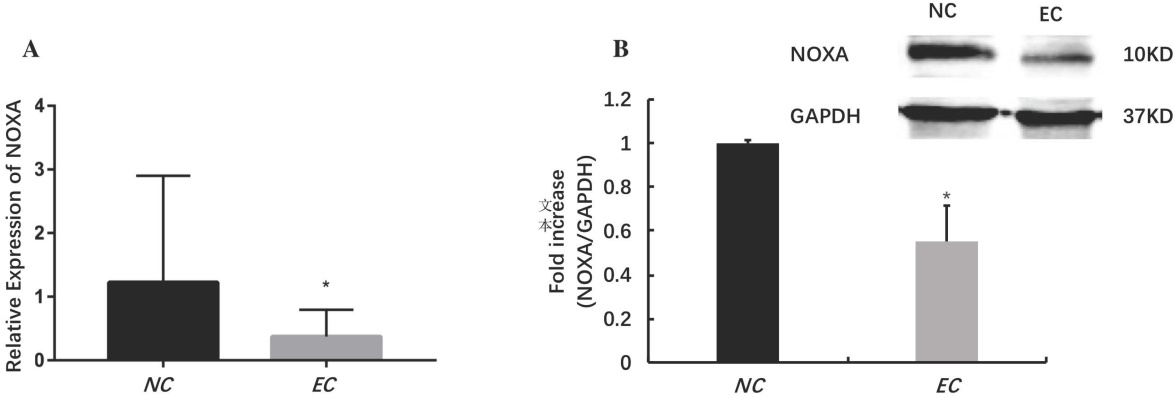
The relative expression levels of noxa in EC tissues (*P<0.05). The mRNA (A) and protein (B) expressions of noxa markedly reduced in EC tissues when compared with NE tissues.

MiRNAs play an important role in regulating the EC. It has identified that microRNA-200c-3p inhibits cell apoptosis by directly targeting noxa in our previous studies 14. This study shows that the expression of microRNA-200c-3p was significantly increased (Figure 1), while the mRNA and protein expressions of noxa remarkably reduced in EC tissues compared with the NE tissues (Figure 2). This suggests that microRNA-200c-3p may regulate the expression of noxa by inducing mRNA cleavage at the post-transcriptional level.

**Figure 3:**
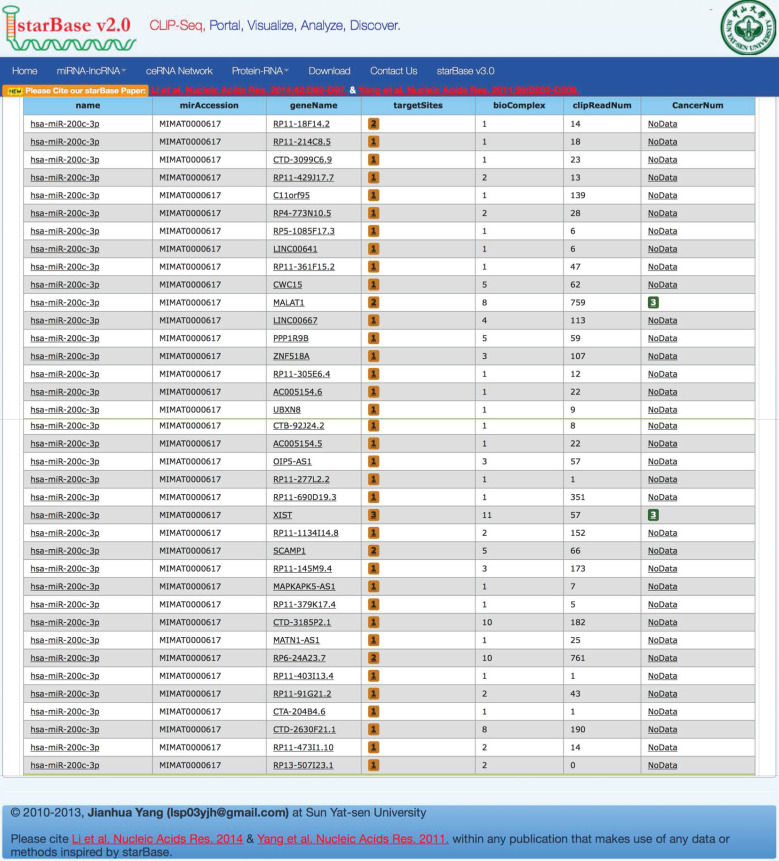
Prediction of lncRNA interacted with microRNA-200c-3p. Using the default parameter settings, starbaseV2.0 identified 37 putative target lncRNAs for microRNA-200c-3p.

**Figure 4:**
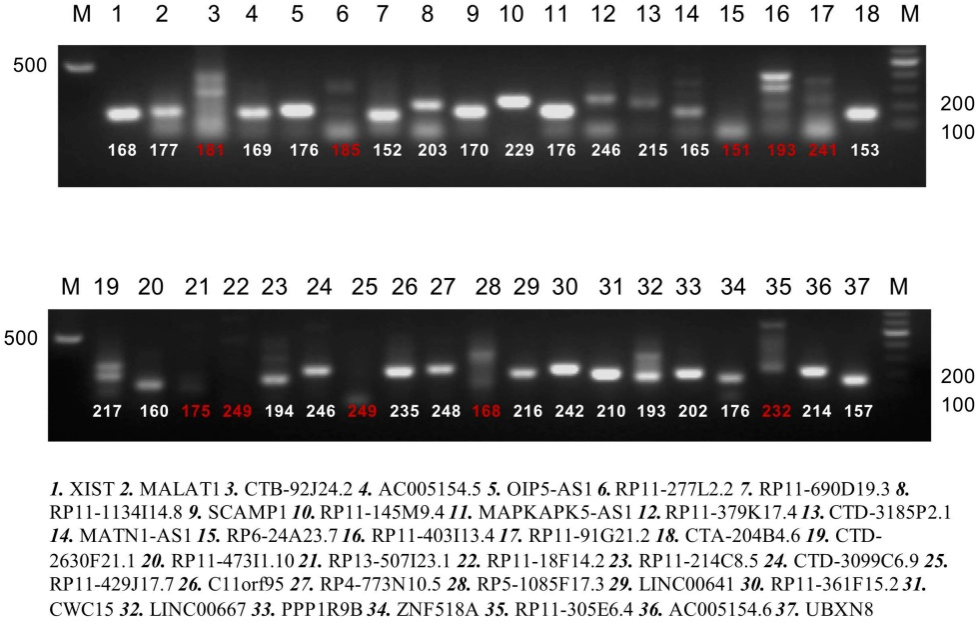
PolyA qualitative analysis of candidate lncRNA (strips marked with red number have not a polyA tail). RT-PCR was performed for these 37 lncRNAs expression and 27 were found to be polyadenylated.

**Figure 5:**
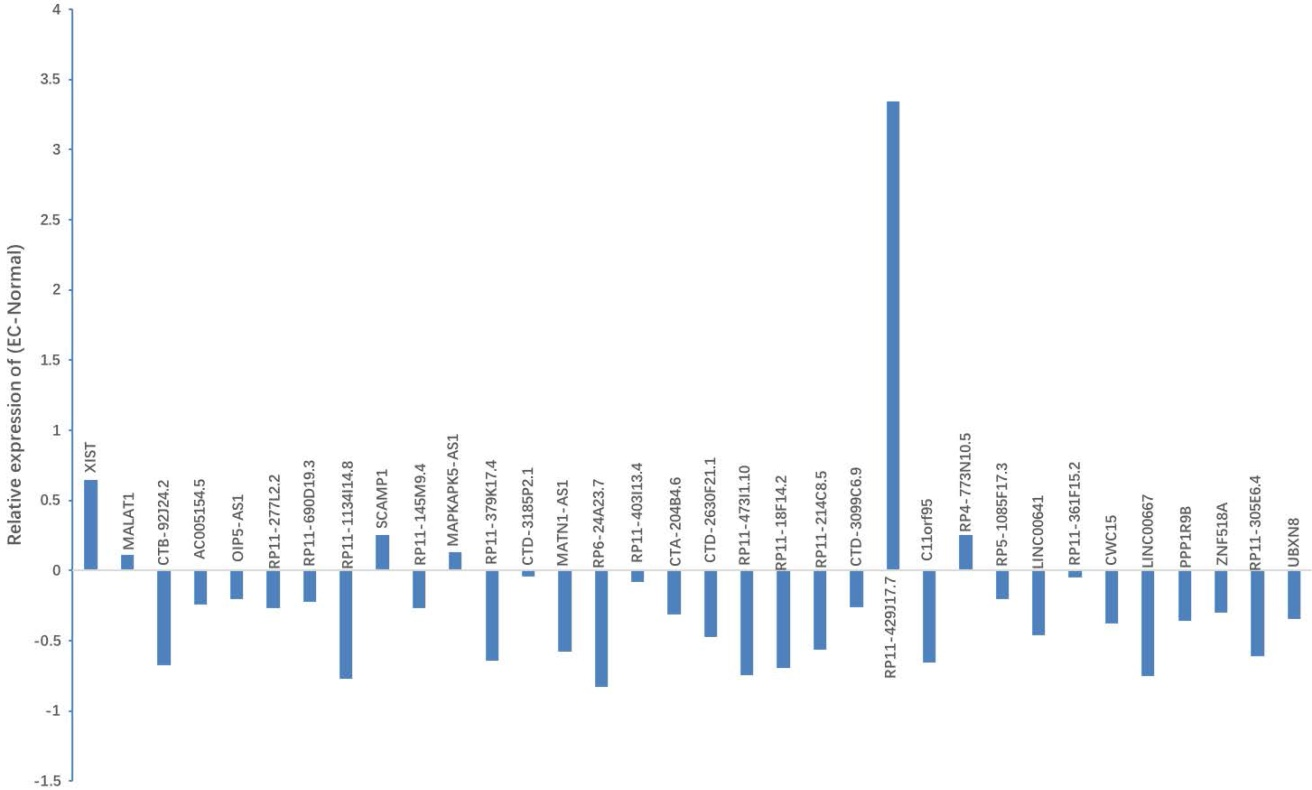
Expression of candidate lncRNA in NE tissues and EC tissues. qRT-PCR was used to detect the expression of 37 lncRNAs. Results indicated that, when compared with NE tissues, 6 lncRNAs displayed up-regulated expression, while 28 lncRNAs showed down-regulated expression in EC tissues. *P < 0.05.

Bioinformatics analysis based on the starbaseV2.0 database was performed to predict the target lncRNA of microRNA-200c-3p. 37 putative target lncRNAs for microRNA-200c-3p were determined while keeping all settings on the default paramet (Figure 3). Subsequently, RT-PCR was made use of the detection of 37 lncRNAs expression, and 27 lncRNAs were revealed to be polyadenylated (Figure 4). Meanwhile, quantitative real-time polymerase chain reaction (qRT-PCR) was performed for detecting the expressions of these lncRNAs. The results showed that 6 lncRNAs in NE tissues were up-regulated, while 28 lncRNAs in EC tissues were down-regulated (Figure 5).

As suggested by the ceRNA hypothesis, the down-regulated ceRNA reduces the cellular concentrations of specific MREs and regulates the inhibition of other transcripts containing the same MREs. On the basis of the noxa expression in EC, we conjectured that it was supposed to have a similar expression profile to noxa in lncRNA with the same MREs of microRNA-200c-3p. According to this hypothesis and the findings mentioned above, 9 lncRNAs with reduced expression were designated as the candidate lncRNAs: RP11-1134I14.8, RP11-379K17.4, MATN1-AS1, CTD2630F21.1, RP11-473I1.10, RP11-214C8.5 and C11orf95, LINC00667 and LINC00641.

### Target relationship between RP11-379K17.4 and microRNA-200c-3p in EC

**Figure 6:**
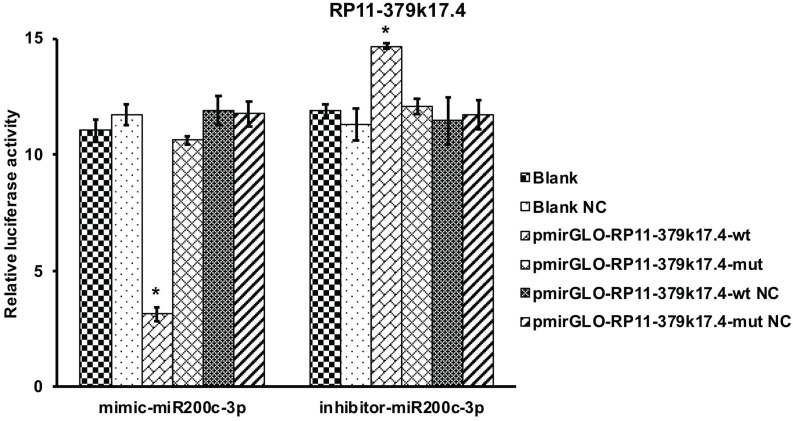
Luciferase assay of candidate lncRNA (*P<0.05). Luciferase assay revealed that microRNA-200c-3p over-expression could significantly reduce the Renilla luciferase activities of RP11-379K17.4.

Bioinformatics analysis revealed that about 9 candidate lncRNAs contained one conservative target site of microRNA-200c-3p. In order to verify the interaction between microRNA-200c-3p and lncRNAs, 9 lncRNAs binding sites were cloned into luciferase reporter PGLO vector. HEK293 cells were co-transfected with miRNAs-200c-3p mimics or inhibitors. Luciferase assay showed that overexpression of microRNA-200c-3p could significantly decrease the Renilla luciferase activity of RP11-379K17.4 (RP11-379K17.4-wt). In addition, the inhibition of microRNA-200c-3p could memorably increase the Renilla luciferase activity of RP11-379K17.4 (RP11-379K17.4-wt) (Figure 6).

By contrast, in the absence of microRNAs-200c-3p target site (RP11-379K17.4-mut), there was no significant change between the cells transfected with PGLO empty vector and RP11-379K17.4 cells (Figure 6), which may be due to the high expression of microRNAs-200c-3p in EC tissues. These results suggest that RP11-379K17.4 may inhibit the expression and activity of microRNA-200c-3p at the post-transcriptional level in normal and cancer tissues through this presumed binding site.

### Expression and clinical significance of RP11-379K17.4 in human EC tissues

The relative expression level of RP11-379K17.4 in the human EC was significantly lower than that in the normal endometrium. The clinicopathological significance of RP11-379K17.4 was evaluated. According to the average expression level of RP11-379k17.4, the patients were divided into high expression group and low expression group. The low expression of RP11-379K17.4 was associated with the late FIGO stage, lymph node metastasis and lymphatic vascular space invasion (P<0.05; Table 1). These results suggest that the decreased expression of RP11-379K17.4 is associated with increased invasive phenotype and metastatic potential.

## Discussion

In recent years, increasingly more proof has shown that lncRNAs are necessary to the development of tumor, and may be prognostic markers and therapeutic targets 24,25. Recently, a newly proposed regulatory mechanism suggests that RNA transcripts can compete to share MRE interactions. Under the circumstances, lncRNAs may act as a target for ceRNA, regulating on sponge miRNAs and applying additional levels of post-transcriptional regulation 20. Researches have shown that the dysregulated ceRNA reciprocity network can facilitate the occurrence and development of multitudinous cancers, involving liver, gastric, colorectal, bladder and endometrial cancers 26-30. For example, lncRNA RP11-395G23.3 and LA16c-313D11.11 act as ceRNA sponges on miR-205-5p to regulate the expression of PTEN in endometrial carcinoma 30.

In our research, it was found that microRNA-200c-3p was observably increased in EC. This is consistent with previous research 22. The lncRNAs containing microRNA-200c-3p binding sites were predicted through Starbase V2.0 online software. Using the polyA qualitative analysis, double luciferase and qRT-PCR assay, we demonstrated for the first time that the expression of lncRNA RP11-379k17.4 was significantly down-regulated in endometrial cancer compared to the normal endometrium, and it acted as a ceRNA on sponge microRNA-200c-3p. Meanwhile, the molecular mechanism and interaction of lncRNA RP11-379k17.4, microRNA-200c-3p and the target gene noxa in EC were first described in our study. Furthermore, we found that the decreased expression of RP11-379K17.4 was associated with increased invasive phenotype and metastatic potential.

Noxa, a pro-apoptotic gene, is a member of the bcl-2 family 6, which contains the BH3 domain and belongs to the subclass of BH3 protein 31. Noxa can exert its pro-apoptotic effect in two ways: on the one hand, the inhibition effect of bcl-2/bcl-xl on Bax/Bak was relieved by combining with the grooves that was formed by noxa's BH3 domain a- helix combination with BH1 and BH2 domain formed on the surface of bcl-2/bcl-xl inhibiting apoptosis on mitochondrial membrane 32-35. On the other hand, the direct interaction with Bax/Bak on the mitochondrial membrane causes conformational changes of Bax/Bak, which leads to cell apoptosis through the mitochondrial pathway 36.Although many studies have demonstrated that noxa is a transcriptional target gene of tumor suppressor gene p53, because it plays a clear role in p53-mediated apoptosis 37,38. Many p53-independent mechanisms of noxa have also been confirmed. Similarly, studies on transcription factor c-Myc, HIF-1a, cAMP response element binding protein (CREB), and E2F transcription factor 1 (E2F1) have also been reported 39-42. In addition, studies have shown that noxa is a direct target of microRNA-200c-3p. Noxa's 3'UTR contains a target site of microRNA-200c-3p. MicroRNA-200c-3p plays an endogenous regulatory role on noxa and inhibits the expression of noxa 14. In this study, we demonstrated that the expression of noxa in endometrial carcinoma was significantly lower than that in normal endometrium.MicroRNA-200c-3p may inhibit the expression of noxa and the apoptosis of endometrial cancer cells by acting on the 3'UTR target site of noxa. We will verify this through further experiments in the future.

In conclusion, we found that RP11-379k17.4 was down-regulated in EC and could release microRNA-200c-3p as ceRNA to inhibit the expression of noxa. RP11-379k17.4 may play a potential role in the progress and development of EC. RP11-379k17.4-microRNA-200c-3p-noxa axis can be used as new clinical markers and therapeutic targets.

## Figures and Tables

**Table 1:** Correlation of RP11-379K17.4 expression with clinicopathological characteristics of EC patients

Variables	Cases (N)	RP11-379K17.4		
		Low (%)	High (%)	*P*
**Age (years)**				0.882
<50	13	7 (15.6)	6 (13.3)	
≥50	32	15 (33.3)	17 (37.8)	
**Histological subtype**				0.823
Endometrioid	36	19 (42.2)	17 (37.7)	
Serous	5	3 (6.7)	2 (4.4)	
Clear cell	4	3 (6.7)	1 (2.2)	
**Menstruation**				0.419
Premenopausal	21	11 (24.4)	10 (22.2)	
Menopausal	24	11 (24.4)	13 (28.9)	
**FIGO stage**				0.047*
Ⅰ	35	15 (33.3)	20 (44.4)	
II	4	2 (4.4)	2 (4.4)	
III	5	4 (8.9)	1 (2.2)	
IV	1	1 (2.2)	0 (0)	
**Histological grade**				0.360
G1	24	11 (24.4)	13 (28.9)	
G2	8	4 (8.9)	4 (8.9)	
G3	4	4 (8.9)	0 (0)	
**Myometrial invasion**				0.233
<1/2	36	16 (35.6)	20 (44.4)	
≥1/2	9	6 (13.3)	3 (6.7)	
**Lymph node metastasis**				0.002*
Present	6	5 (11.1)	1 (2.2)	
Absent	39	17 (37.8)	22 (48.9)	
**Lymphovascular space invasion**				0.009*
Present	9	8 (17.8)	1 (2.2)	
Absent	36	14 (31.1)	22 (48.9)	

*P < 0.05.
